# Altered Serum IgG Levels to α-Synuclein in Dementia with Lewy Bodies and Alzheimer’s Disease

**DOI:** 10.1371/journal.pone.0064649

**Published:** 2013-05-31

**Authors:** Niklas K. U. Koehler, Elke Stransky, Mona Shing, Susanne Gaertner, Mirjam Meyer, Brigitte Schreitmüller, Thomas Leyhe, Christoph Laske, Walter Maetzler, Phillipp Kahle, Maria S. Celej, Thomas M. Jovin, Andreas J. Fallgatter, Anil Batra, Gerhard Buchkremer, Klaus Schott, Elke Richartz-Salzburger

**Affiliations:** 1 Department of Psychiatry and Psychotherapy, Eberhard-Karls-University, Tübingen, Germany; 2 Department of Neurodegeneration, Hertie Institute for Clinical Brain Research, Tübingen, Germany; 3 Functional Neurogenetics, Hertie Institute for Clinical Brain Research, Tübingen, Germany; 4 Max-Planck-Institute for Biophysical Chemistry, Göttingen, Germany; Philadelphia VA Medical Center, United States of America

## Abstract

Natural self-reactive antibodies in the peripheral blood may play a considerable role in the control of potentially toxic proteins that may otherwise accumulate in the aging brain. The significance of serum antibodies reactive against α-synuclein is not well known. We explored serum IgG levels to monomeric α-synuclein in dementia with Lewy bodies (DLB) and Alzheimer’s disease (AD) with a novel and validated highly sensitive ELISA assay. Antibody levels revealed stark differences in patients compared to healthy subjects and were dependent on diagnosis, disease duration and age. Anti-α-synuclein IgG levels were increased in both patient groups, but in early DLB to a much greater extent than in AD. Increased antibody levels were most evident in younger patients, while with advanced age relatively low levels were observed, similar to healthy individuals, exhibiting stable antibody levels independent of age. Our data show the presence of differentially altered IgG levels against α-synuclein in DLB and AD, which may relate to a disturbed α-synuclein homeostasis triggered by the disease process. These observations may foster the development of novel, possibly preclinical biomarkers and immunotherapeutic strategies that target α-synuclein in neurodegenerative disease.

## Introduction

Alzheimer’s disease (AD) and dementia with Lewy bodies (DLB) are the most frequent forms of dementia and belong to the tauopathies and synucleinopathies, respectively. Clinical diagnosis and distinction of the disorders are often difficult especially at very early and late stages of disease, with a definite diagnosis usually only being made upon autopsy. Despite the revised clinical guidelines, the consensus criteria for the diagnosis of DLB [1] is frequently lacking sensitivity, especially at disease onset [2]. In addition, there is considerable clinical and pathological overlap between the two disorders [3]. In AD, Lewy bodies and Lewy neurites are frequently observed [4], and DLB often meets the histological criteria of AD [1]. Therefore, early, preferably preclinical diagnosis of an evolving dementia and a better diagnostic distinction of DLB versus AD is an important goal. To meet this challenge, the development of sensitive and specific biomarkers is crucial.

The biochemical markers Amyloid beta (Aβ) 1–42 and tau in the cerebrospinal fluid (CSF), that are decreased and elevated in AD, respectively, offer reasonable sensitivity and specificity for the diagnosis of AD, especially if analyzed together, however, their ability to differentiate AD from other forms of dementia, such as DLB, is limited [5], [6]. Increasingly, α-synuclein, a protein involved in synaptic plasticity and neurotransmission, is being explored as an additional CSF biomarker, and evidence may point to lower levels in Parkinson’s disease (PD) patients compared to controls [5], [7]–[9]. The presence of antibodies against α-synuclein in the peripheral blood was first published by Woulfe et al. 2002 [10] and later by Papachroni et al. 2007 [11] but no significant differences of antibody levels were observed in idiopathic PD compared to controls. However, Gruden et al. detected elevated antibody levels against α-synuclein in patients with PD [12]–[14]. This is in contrast to recent observations by Besong-Agbo et al. describing decreased anti-α-synuclein antibody levels in PD but not AD patients compared to healthy controls [15]. Very recently, Smith et al. observed increased serum antibody concentrations within the PD patient group only if disease duration was less than 4 years [16].

Alpha-synuclein is an abundant self-protein and is found at low concentration extracellularly in serum and CSF and intracellularly at relatively higher concentration in neurons and blood cells [17], [18]. Alpha-synuclein can endogenously be transferred from neurons to astroglial cells and may promote a local inflammatory response [19]. Moreover, it was recently recognized that prion-like spread of α-synuclein aggregates into neurons may lead to Lewy body pathology and neuronal loss in PD [20]–[23]. Importantly, in the mouse model Lewy body pathology could be prevented by active immunization with α-synuclein or direct transfer of antibodies against α-synuclein [24], [25].

The natural antibody repertoire against α-synuclein is likely being shaped by positive B cell selection and α-synuclein presentation to lymphocytes. In neurodegenerative disease blood-borne monocytes/macrophages or activated CNS (micro)glia may act as antigen-presenting cells (APC) [26], [27] and drive B cell activation and selection, resulting in production of affinity-matured antibodies to α-synuclein. Therefore, delineation of anti-α-synuclein antibodies in healthy and diseased individuals could give important insight for the discovery of novel immune biomarkers and, in addition, for the development of α-synuclein targeted immunotherapies. In this study, we measured IgG levels against α-synuclein in the serum of DLB, AD and non-demented control subjects and analyzed their relation to other demographic factors, such as age at blood draw, gender, disease duration and severity.

## Materials and Methods

### Patients and Controls

The ethics committee of the Eberhard-Karls-University Tübingen approved this study (ethics nr. 314/06) and written informed consent was received by all individuals participating in the study. If capacity to consent of participants was compromised by any means, based on neuropsychological testing and physicians’ judgment, legal guardians consented on their behalf. All participants who declined to participate or otherwise did not participate in the study were not disadvantaged in any other way.

Serum samples were collected at the Department of Psychiatry and Psychotherapy and the Department of Neurodegeneration, Eberhard-Karls-University Tübingen. A total of 19 DLB patients, 15 AD patients and 16 age-matched, non-demented controls (C) were investigated. AD patients met the diagnostic criteria of probable AD according to DSM-4, ICD-10 and NINCDS-ADRDA (National Institute of Neurological and Communicative Disorders and Stroke-Alzheimer’s Disease and Related Disorders Association). DLB was diagnosed according to the criteria of McKeith et al. [1] with central features, and at least one core feature being present. Severity of dementia was assessed by mini-mental state examination (MMSE) [28]. Patients with presence of cerebrovascular disease on brain imaging, a physical or brain disorder that could account for the clinical picture were excluded. Healthy control individuals were recruited from healthy spouses or volunteers that did not complain of any cognitive problems, and who were unremarkable in cognition and cognitive flexibility.

### Data and Statistical Analysis

Data are shown as mean ± standard deviation (SD). Statistical evaluation and regression analysis were carried out as indicated using the GraphPad Prism 5 software (La Jolla, CA; USA). Groups were compared by unpaired or paired two tailed t-test, as indicated. Analysis of multiple diagnostic groups was performed with one-way ANOVA followed by Tukey’s multiple comparison test. Demographics were analyzed by unpaired two-tailed t-test, one-way ANOVA or χ2-test, as appropriate. Correlations were assessed by Spearman correlation. Receiver operating characteristic (ROC) curves, along with area under the curve (AUC) and p-values were generated with GraphPad Prism. The significance level was generally set at p<0.05 (***p<0.001, highly significant; **p = 0.001 – 0.01, very significant; *p = 0.01 – 0.05, significant; >0.05 not significant).

### Measurement of IgG Antibodies against α-synuclein

Serum IgG levels against α-synuclein were measured by a sandwich ELISA developed in our laboratory as described below. Alpha-synuclein produced in E. coli was analyzed for purity by Western immunoblot (not shown). 96 well plates (Polysorb, Nunc, Wiesbaden, Germany) were coated with 3 µg/ml recombinant human α-synuclein in TBS buffer and incubated overnight at 4°C. Plates were washed in TBS/Tween (TBS, 0.1% Tween) and blocked in TBS/Tween plus proprietary proteins. Duplicate wells were incubated with serum diluted in TBS/Tween for 2 hours. After washing, wells were incubated with biotinylated polyclonal goat anti-human IgG (Sigma-Aldrich, Deisenhofen, Germany) diluted 1∶5000 in TBS/Tween. Avidin-Biotin (Roche, Mannheim, Germany) was used as an amplification step and TMB as the peroxidase substrate. The colour reaction was stopped after 30 minutes. Absorbance (OD, optical density) as a measure of antibody reactivity or antibody level was measured at 450nm in an ELISA reader (Sunrise, Tecan, Switzerland). Corrected specific IgG reactivity against α-synuclein was calculated by subtraction of unspecific background signal (α-synuclein coated wells, serum omitted) from specific signal (serum incubation). A monoclonal mouse antibody to anti-α-synuclein (clone Syn211, Sigma-Aldrich, Deisenhofen, Germany) served as coating control. Specificity of measured anti-α-synuclein IgG was further demonstrated in a competition (blocking) assay by pre-incubation of serum with recombinant α-synuclein prior to ELISA analysis. In addition, a solid phase α-synuclein pre-adsorption assay by means of a well to well serum transfer was performed (not shown).

## Results

### Patient Population and Demographics

Details of the population demographics are shown in **Table 1**. Disease duration, age at disease onset and MMSE was not significantly different between DLB and AD by unpaired two tailed t-test with p-values of 0.86, 0.09 and 0.88, respectively. Age did not differ significantly between the groups including the non-demented controls (C) by one-way ANOVA (p = 0.08), while gender was significantly different between all groups by χ2-test (p = 0.018), due to the high prevalence of female patients in the AD group.

**Table 1:** Demographics and anti-α-synuclein IgG levels of DLB, AD and control (C) group.

	n (f/m)	Age (y)	Diseaseduration (y)	Age at disease onset (y)	MMSE (score)	anti-α-syn IgG (OD)
**C**	16 (9/7)	70.9±4.6	–	–	n.d.	0.484±0.245 (CI 0.354–0.615)
**DLB**	19 (6/13)	75.1±7.7	3.4±2.8	72.5±7.3	18.5±7.7	0.801±0.484 (CI 0.568–1.034)
**AD**	15 (12/3)	70.3±7.1	3.3±2.2	67.9±8.0	18.8±5.5	0.751±0.363 (CI 0.551–0.952)

Data are shown as mean ± SD. AD = Alzheimer’s disease; C = non-demented controls; CI = 95% confidence interval of the mean; DLB = dementia with Lewy bodies; f = female; m = male; MMSE = Mini-mental state examination; n = number of individuals; n.d. = not done; OD = optical density; syn = synuclein; y = years.

### Measurement of Anti-α-synuclein IgG

The ELISA was extensively tested with various modifications to achieve utmost specificity and reliability. After testing a wide range of serum dilutions (not shown) a dilution of 1∶100 was chosen, because at this dilution measured IgG levels fell mostly within the range of the assay and were above the detection limit (unspecific background +3SD). The linear, dynamic range of the assay was from approx. 0.8 to 2.5 OD. Measurements were replicated in at least 3 independent assays using the same sera (not shown), and interassay variation was 30% at a maximum. Yet, to reach the highest consistency for a reliable comparison of anti-α-synuclein IgG levels, only data are shown that were generated within the same experimental run. The unspecific background staining (α-synuclein coated wells, only serum omitted) was very low with an OD of 0.067±0.018 (mean ± SD). The anti-α-synuclein coating control antibody (clone Syn211) yielded ODs of 2.61±0.13, 1.25±0.12 and 0.56±0.02 at dilutions of 1∶50′000, 100’000 and 500’000, respectively.

### Blocking of Serum Anti-α-synuclein IgG Antibodies

**Figure 1:**
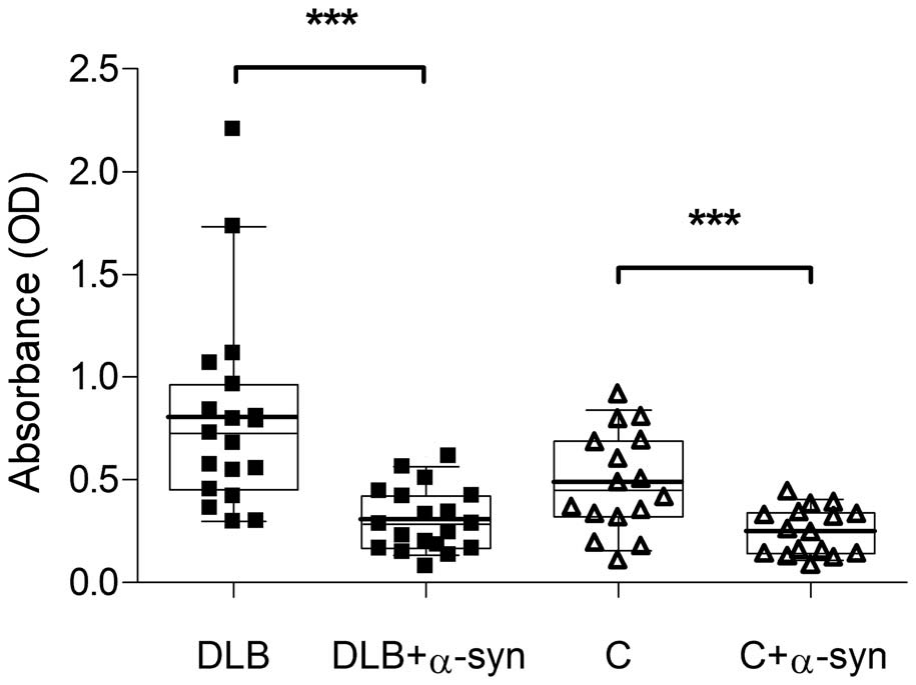
Blocking of serum anti-α-synuclein IgG antibodies. Pre-incubation of serum from DLB patients and non-demented controls (C) with recombinant human α-synuclein (α-syn) efficiently bound serum IgG against α-synuclein, indicating the specificity of the ELISA measurements. Anti-α-synuclein IgG reactivity from DLB and controls (C) was blocked by an average of 55.8 and 43%, respectively. The boxes upper and lower borders represent the 25th and 75th percentile, whiskers indicate the 10 to 90 percentile, and the solid line within the box marks the mean, the thin line the median of the data points. p<0.0001 by paired two-tailed student t-test.

To validate the specificity of anti-α-synuclein IgG measurements, serum was pre-incubated at 20°C for 1 hour with recombinant human α-synuclein at 5 µg/ml in order to bind free serum antibodies against α-synuclein. Pre-treated sera from the 19 DLB patients and the 16 non-demented control individuals (not performed for AD samples) were subsequently measured by ELISA, shown in **Fig. 1**. The addition of α-synuclein resulted in diminished anti-α-synuclein IgG levels in all sera. Antibodies to α-synuclein were efficiently reduced to a mean absorbency of 0.30 OD (from 0.80) and 0.29 OD (from 0.48) in DLB and controls, respectively. The reduction of anti-α-synuclein reactivity was highly significant for both groups (p<0.0001) by paired two-tailed student t-test, indicating the specificity of the ELISA.

### Serum Levels of Anti-α-synuclein IgG in DLB, AD and Controls

**Figure 2:**
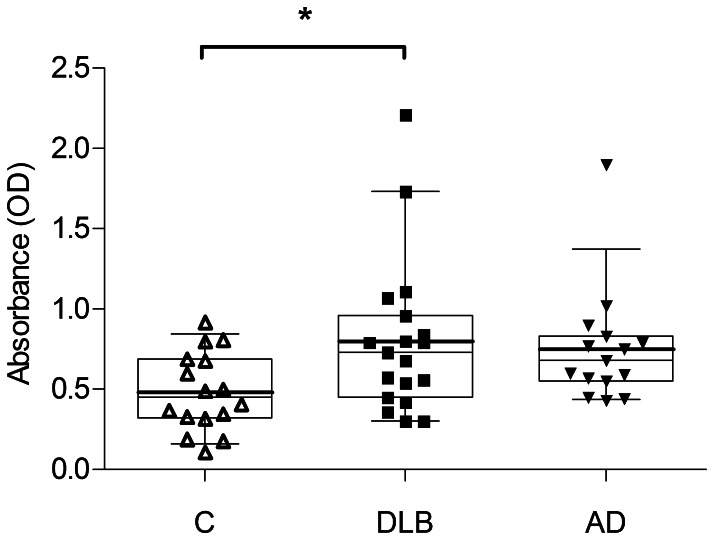
Serum levels of anti-α-synuclein IgG in DLB, AD and controls. Comparison of serum IgG levels against α-synuclein in DLB, AD and control group (C). By ANOVA there was a significant difference between the groups (p = 0.0470) and anti-α-synuclein IgG was significantly higher in DLB compared to non-demented controls (p<0.05) by Tukey’s multiple comparison test.

DLB and AD patients revealed elevated mean serum IgG levels against α-synuclein compared to non-demented control individuals, shown in **Fig. 2**, while mean antibody levels were similar in DLB and AD patients. The difference of IgG levels between the three groups was significant by ANOVA (p = 0.0470) and DLB significantly differed from controls by Tukey’s multiple comparison test (p<0.05). Very high antibody levels above 1 OD were only observed in the patient groups. An OD greater than 0.7 was observed in 10 of 19 (52.6%) DLB patients and 7 of 15 (46.7%) AD patients. By contrast, only 3 of 16 (18.8%) controls reached that level. Although IgG levels considerably overlapped between the groups, ODs greater than 1 were only observed in patients (n = 4) and ODs below 0.43 essentially in controls (n = 8) and only one DLB patient. It is of note that the distribution of IgG levels against α-synuclein was spread out much more in DLB and AD than controls, reflected by higher standard deviations/variances of mean IgG levels in the patient groups (**Tab. 1**). The 95% confidence interval (CI) of the mean was 0.354–0.615 OD, 0.568–1.034 OD and 0.551–0.952 OD for controls, DLB and AD patients, respectively.

### Influence of Age on Serum Anti-α-synuclein Antibody Levels

**Figure 3:**
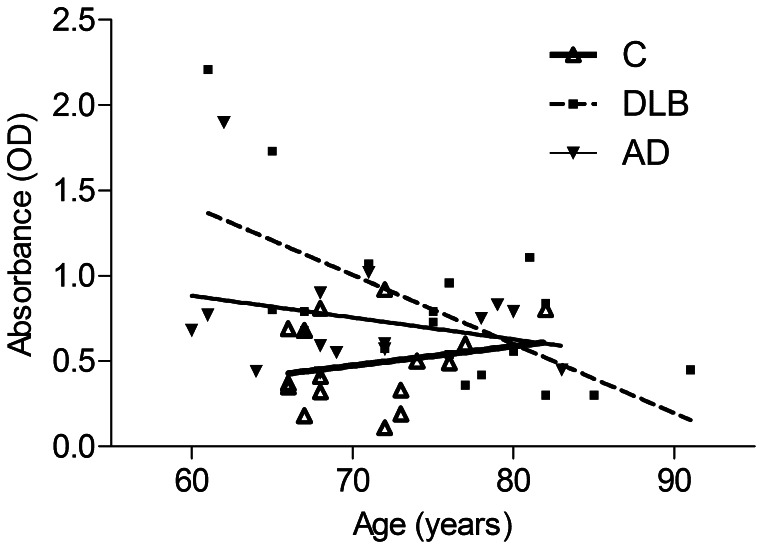
Influence of age on serum anti-α-synuclein antibody levels. Linear regression of serum anti-α-synuclein IgG levels with age for the DLB, AD and control group. The difference between the slopes was significant (p = 0.0385). DLB and to a lesser extent AD showed noticeably higher antibody levels at younger age compared to patients with advanced age. The negative correlation of anti-α-synuclein IgG levels with age in DLB patients was highly significant (p = 0.0074 by Spearman correlation). By contrast, the control group showed a trend of rising IgG levels with age.

Linear regression of age with IgG levels to α-synuclein is illustrated in **Fig. 3** for the DLB, AD and the non-demented control group (C). For DLB patients correlation of anti-α-synuclein IgG levels with age was strongly negative and highly significant by Spearman correlation p = 0.0074. Thus, serum IgG levels were significantly higher in younger compared to DLB patients with advanced age. AD patients showed a negative, but non-significant trend of IgG levels with age and, by contrast, controls exhibited slightly rising IgG levels with age. R square was 0.0048, 0.421, 0.063 for controls, DLB and AD, respectively. Only for DLB the slope deviated highly significant from zero (p = 0.0027). Until the age of about 75 years, DLB and AD patients exhibited considerably higher anti-α-synuclein IgG levels that were all above the regression line of the control group; this difference between patients and controls diminished at a higher age.

### Influence of Disease Duration on Serum Anti-α-synuclein IgG Levels

**Figure 4:**
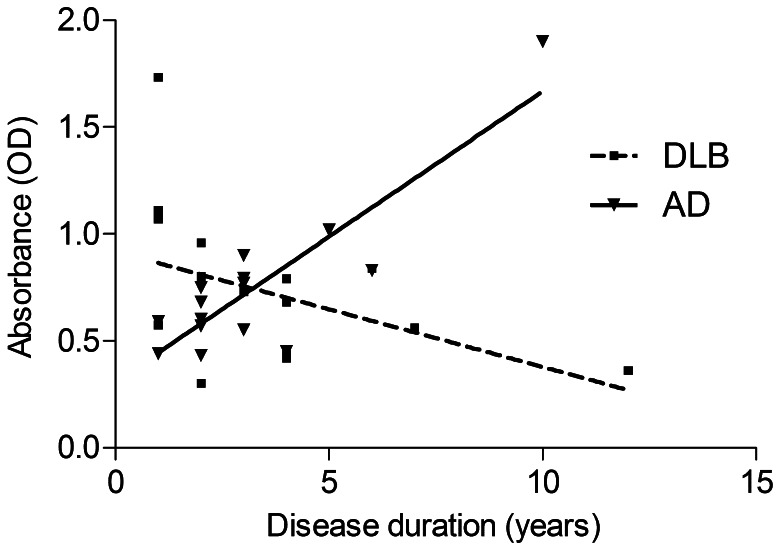
Influence of disease duration on serum anti-α-synuclein IgG levels. Linear regression of serum anti-α-synuclein IgG levels with duration of disease for DLB and AD patients. In AD longer disease was strongly correlated with higher antibody levels (p = 0.010 by Spearman correlation). In contrast, in DLB patients, disease duration and anti-α-synuclein IgG levels showed a tendency of negative correlation (p = 0.056).

In DLB and AD patients linear regression of disease duration and serum anti-α-synuclein IgG levels showed associations in opposite direction, shown in **Fig. 4**. There was a strong positive relationship between disease duration and anti-α-synuclein IgG levels in AD, reaching a p-level of 0.010 by Spearman correlation. By contrast, in DLB longer disease span was linked to lower IgG levels, with highest levels observed at very early stages (p = 0.056 by Spearman correlation). The slope deviated highly significant from zero for AD (p<0.0001), but not for DLB (p = 0.090). The difference between the slopes of both patient groups was highly significant (p<0.0001). R square was 0.752 and 0.180 for AD and DLB, respectively.

### Influence of Gender and MMSE on Serum Anti-α-synuclein IgG Levels

There was no evidence of higher antibody levels of females compared to males in DLB or controls (not shown). No significant correlation was observed between disease severity (MMSE) and anti-α-synuclein antibody levels in the patient groups (not shown).

### ROC Curve Analysis to Evaluate Anti-α-synuclein IgG Levels as a Marker of Disease

**Figure 5:**
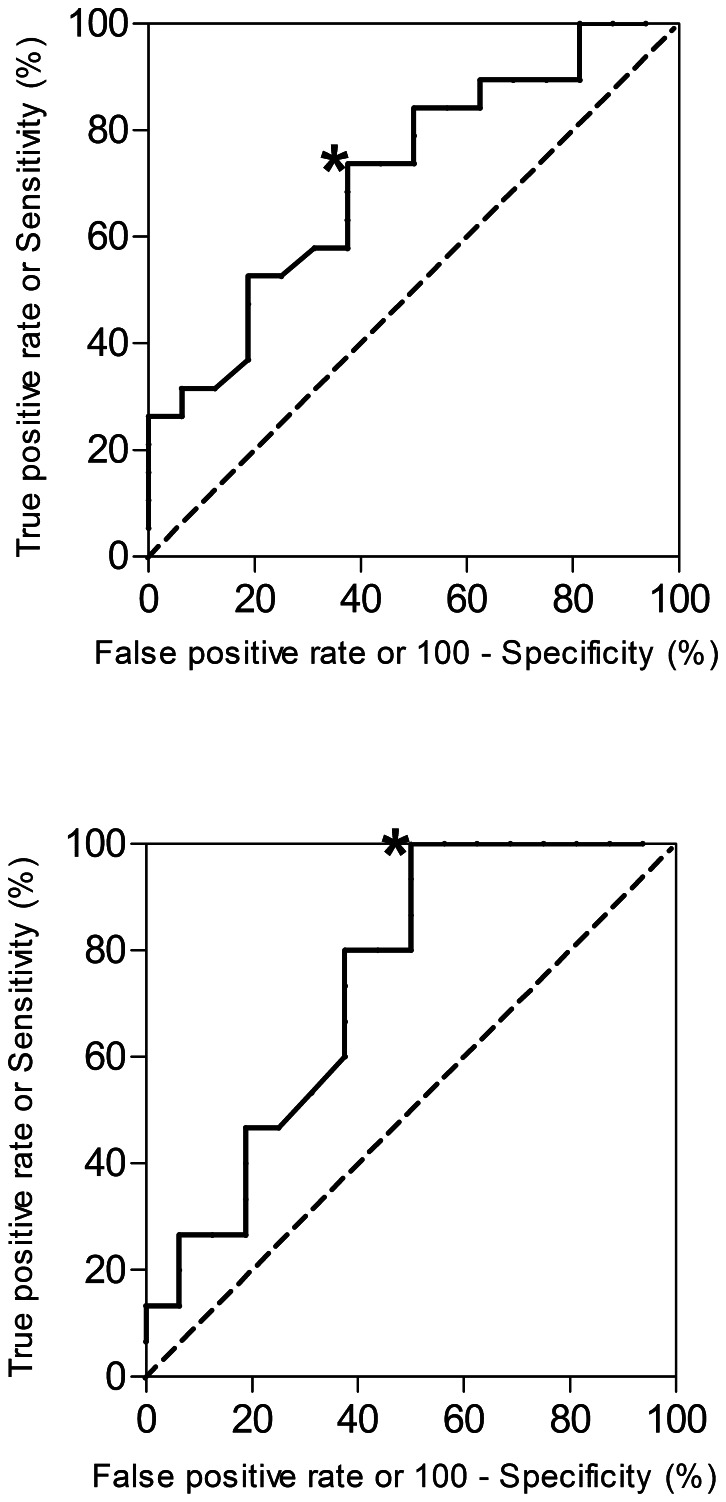
ROC curve analysis to evaluate anti-α-synuclein IgG levels as a marker of disease. Receiver-operator characteristic (ROC) curves to evaluate antibody levels against α-synuclein as a diagnostic biomarker for DLB or AD. Anti-α-synuclein IgG levels were used to create ROC curves to differentiate DLB from controls (AUC = 0.714; p = 0.031) (Fig. 5A) and differentiate AD from controls (AUC = 0.738, p = 0.024) (Fig. 5B). No diagnostic value was reached for the differentiation of AD from DLB (not shown). The dashed line indicates the threshold of no diagnostic value (AUC = 0.5). Youden index was calculated to identify cut-off levels, denoted by the star.

Receiver operating characteristic (ROC) curve analysis was utilized to evaluate the potential of anti-α-synuclein IgG levels to indicate disease, or to differentiate between DLB (**Fig. 5A**) and AD (**Fig. 5B**). The ROC curve plots the rate (in percent) of true positive (sensitivity) against false positive (specificity) diagnosis (C vs. DLB or AD) for every anti-α-synuclein IgG cut-off value. Diagnostic usefulness of the ROC curves was confirmed by the area under the curve (AUC), which was 0.714 (95% CI: 0.543–0.884; p = 0.031) and 0.738 (95% CI: 0.560–0.916; p = 0.024) for DLB and AD vs. controls, respectively. ROC analysis did not indicate significance for the discrimination between AD and DLB patients. The dashed line (AUC = 0.5) in Figures 5 indicates the threshold where there would be no diagnostic value to anti-α-synuclein IgG levels. The highest predictive value would be reached when AUC = 1. The maximum Youden index which describes the performance of a diagnostic test/biomarker was used to determine the potentially optimal anti-α-synuclein IgG cut-off level, marked by a star in Fig. 5A and Fig. 5B, to separate AD or DLB patients from controls. The cut-off levels that may represent the best balance between sensitivity and specificity were at 0.52 OD for DLB, resulting in 74% sensitivity and 63% specificity, and 0.42 OD for AD, resulting in 100% sensitivity, 50% specificity.

## Discussion

To correctly diagnose an evolving, potentially dementing neurodegenerative disorder poses a great challenge even to the specialized physician. This difficulty is particularly apparent at the very early and late stages of disease, when neuropsychiatric symptoms are subtle or intertwine. The great majority of these disorders are characterized by tissue deposition of the aggregation prone and ubiquitously expressed proteins Aβ, tau and α-synuclein [29]–[31]. In view of the immensely growing economic burden that these disorders pose onto the ageing society and with regard to the right use of existing and emerging drug therapies, an earlier and a more precise diagnosis is urgently needed.

Alpha-synuclein plays a key role in the pathogenesis of synucleinopathies, which comprise PD, PD dementia (PDD), DLB and multiple system atrophy (MSA), in addition to AD subtypes, such as the Lewy body variant of AD. In these disorders pathological conformations of α-synuclein aggregate, accumulate and deposit in the nervous system in the form of oligomers or insoluble amyloid [32]–[34]. Because of the relative abundance of activated cytokine producing APCs such as microglia and macrophages in afflicted nervous system tissue [26], [27], it is reasonable to assume that an adaptive immune response would result in the production of antibodies against α-synuclein, which may ideally be characteristic for a particular synucleinopathy.

We developed and validated a highly sensitive and specific ELISA to measure antibody reactivities against monomeric α-synuclein in human serum. Intriguingly, antibody levels were differentially altered in DLB and AD compared to healthy control subjects. In young patients serum IgG levels were strikingly elevated in DLB and to a lesser extent also in AD and markedly decreased with advancing age. By contrast, anti-α-synuclein antibodies from controls remained stable at lower levels throughout the aging process. Notably, antibody production was also dependent on disease duration; DLB patients exhibited very high IgG levels at early stages, whereas AD patients showed the reverse, with higher levels after a prolonged disease course. Anti-α-synuclein antibodies may be triggered by an inflammatory CNS microenvironment, frequently present in AD and PD [26], [27], [35], [36] and related to the amount of α-synuclein aggregation and tissue deposition, which is likely extensive in early DLB, in contrast to AD where α-synuclein burden may slowly rise. The steep drop of the elevated α-synuclein-reactive antibody levels with age in DLB and AD may be due to absorption of antibodies by tissue-deposited α-synuclein and, in addition, pathophysiologically related to an accelerated immunosenescence with a disturbed T helper (Th) lymphocyte adaptive immunity [36]–[40]. Based on these observations anti-α-synuclein antibodies may represent a powerful immune biomarker for the detection of an ongoing, yet preclinical α-synuclein associated neurodegeneration, and be of significant help for the diagnosis of (pre)symptomatic disease, potentially indicating the load of Lewy-body pathology. It seems reasonable to hypothesize that an emerging α-synucleinopathy could be revealed by increasing anti-α-synuclein IgG titers.

In this context it should be noted that according to our analysis intergroup differences of anti-α-synuclein IgG levels between DLB and AD seem modest and specificity and sensitivity are at this stage not sufficient for a clinical discriminator. Also, a confirmation dataset is needed to substantiate our findings. Similar changes may occur in subjects with other prototypic synucleinopathies such as PD and MSA which was not part of this study.

So far, only a few studies analyzed antibody reactivities against α-synuclein in PD. Their presence was first identified in serum by ELISA, however, there was no difference of antibody levels in PD compared to control individuals [10]. In a Western blot analysis serum antibodies against α-synuclein were detected in the majority of patients with inherited PD, but the difference between idiopathic PD and control subjects was not statistically significant [11]. It was only recently, that higher IgG levels against α-synuclein were described in PD especially early in the disease [12]–[14], [16] which was not confirmed in another study, showing lower antibody levels [15]. In comparison, Aβ the major amyloid that accumulates in AD plaques has been studied more extensively. Altered autoantibody reactivities against Aβ have been detected in AD, showing increased, decreased or similar antibody levels in patients compared to controls in CSF [41] and plasma or serum [41]–[48]. This conflicting data may be not only explained by methodological differences of the employed immunoassays but also by other influencing variables, such as age and disease duration as observed in our study populations.

The pathophysiological relevance of autoantibodies that bind to α-synuclein is unknown. Elevated antibody levels could play a role for both, a beneficial immune response as part of a protective mechanism against the accumulation of pathogenic α-synuclein or, but probably less likely, a harmful inflammatory process that mediates CNS damage. Analogous to the proposed homeostasis of Aβ concentrations between CNS and peripheral circulation by anti-Aβ antibodies (peripheral sink hypothesis) [33], [49]–[51], antibodies to α-synuclein may draw α-synuclein into the peripheral circulation [52], and thereby clear the protein or its toxic intermediates from the CNS in order to maintain a physiological equilibrium. Antibodies could lead to immune-complex formation, mediate phagocytosis by macrophages/microglia [53], or have a direct proteolytic activity [54] and control potentially toxic protein levels. Monoclonal antibodies against α-synuclein have been shown to reduce oligomerization and increase turnover of α-synuclein [55]. Thus, antibodies to α-synuclein may critically influence concentration and half-life of α-synuclein in body fluids and CNS tissue and act as chaperones to prevent α-synuclein from forming toxic or aggregation prone conformations.

There is strong evidence of a transneuronal prion-like spread of misfolded amyloidogenic proteins such as α-synuclein through the extracellular compartment in neurodegenerative disease [20]–[23]. Therefore, therapeutic targeting of extracellular α-synuclein by passive antibody administration, an approach currently extensively pursued for Aβ in AD clinical trials, appears feasible. In synucleinopathies and in particular DLB this could be most promising also later in the disease course when anti-α-synuclein IgG levels are diminished.

Antibodies to self-proteins such as α-synuclein or Aβ can be readily detected in the peripheral blood of healthy individuals and are a normal part of the antibody repertoire. In contrast to the former theory of deletion of autoreactive clones, “horror autotoxicus” as coined by Paul Ehrlich, it is now anticipated that self-reactive antibodies are playing an important role for the biochemical and immunological homeostasis [56], [57]. These natural autoantibodies in healthy individuals may serve a protective function by binding endogenous, perhaps toxic proteins or by neutralizing microbes as part of a first defence barrier throughout life [58], [59].

Taken together, the presence of α-synuclein-specific antibodies in the peripheral blood points to their potential relevance for maintaining equilibrium of α-synuclein or its degradation products within the CNS and the peripheral circulation. Based on our results, the analysis of antibody reactivities against α-synuclein may provide a means to diagnose and assess neurodegenerative diseases where α-synuclein plays a pathophysiological role. Moreover, the detection of elevated antibody levels against α-synuclein could be a valuable predictor for early stage Lewy body disease, especially in younger individuals. Thus, further exploration of anti-α-synuclein antibodies as a potential diagnostic biomarker for α-synuclein related neurodegenerative disease is warranted.
